# Ca^2+^ Influx in T Cells: How Many Ca^2+^ Channels?

**DOI:** 10.3389/fimmu.2013.00099

**Published:** 2013-04-24

**Authors:** Stefan Feske

**Affiliations:** ^1^Department of Pathology, New York University Langone Medical CenterNew York, NY, USA

Ca^2+^ signals are critical for T cell function. A number of ion channels regulate Ca^2+^ influx from the extracellular space in T cells, either by conducting Ca^2+^ ions or by modulating the membrane potential that provides the driving force for Ca^2+^ influx (Cahalan and Chandy, 2009; Feske et al., 2012). The best characterized Ca^2+^ channel in T cells is the Ca^2+^ release-activated Ca^2+^ (CRAC) channel, which mediates store-operated Ca^2+^ entry (SOCE) in response to T cell receptor (TCR) activation and is composed of ORAI and stromal interaction molecules (STIM) family proteins. Several other channels may also mediate Ca^2+^ influx directly in T cells including members of the transient receptor potential (TRP) family, P2X receptors, and voltage-gated Ca^2+^ (Ca_v_) channels. Compared to CRAC channels, however, their contribution to TCR-induced Ca^2+^ influx and immunity is less well defined.

Ca^2+^ release-activated Ca^2+^ channels were first identified in T cells (and mast cells) over 20 years ago (Lewis and Cahalan, 1989; Hoth and Penner, 1992; Zweifach and Lewis, 1993). They mediate Ca^2+^ influx and have well defined electrophysiological properties (Parekh and Penner, 1997; Prakriya and Lewis, 2003). The long elusive molecular identity of the CRAC channel was solved with the discovery of ORAI1 by genome-wide RNAi screens and positional cloning in patients lacking CRAC channel function (Feske et al., 2006; Vig et al., 2006b; Zhang et al., 2006). ORAI1 and its two homologs, ORAI2 and ORAI3, are integral membrane proteins (Figure 1). Mutagenesis and structural analyses have showed that ORAI1 forms the pore of the CRAC channel through which Ca^2+^ ions enter the cell (Prakriya et al., 2006; Vig et al., 2006a; Yeromin et al., 2006; Hou et al., 2012; McNally et al., 2012). CRAC channels open after TCR-induced production of inositol 1,4,5-trisphosphate (InsP3) and release of Ca^2+^ from ER stores. Reduced Ca^2+^ levels in the ER trigger the activation of STIM 1 and 2 located in the ER membrane. After translocation to ER-plasma membrane junctions, STIM proteins bind to ORAI1 and open the CRAC channel pore, resulting in sustained Ca^2+^ influx. The molecular regulation of CRAC channel function has been described in detail elsewhere (Shaw et al., 2012).

**Figure 1:**
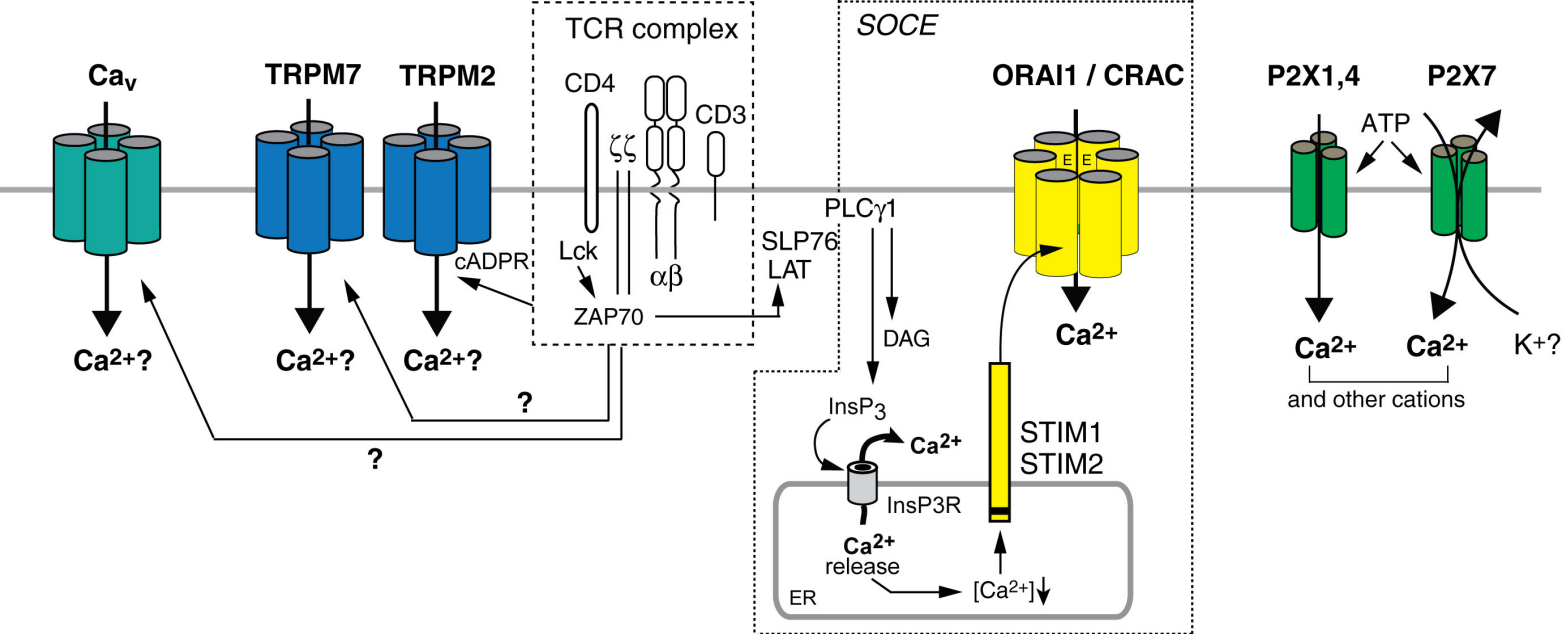
**Ca^2+^ influx pathways in T cells**. Stimulation of T cells through the TCR complex results in Ca^2+^ influx, which is involved in the regulation of many T cell functions. CRAC channels mediate store-operated Ca^2+^ entry (SOCE) following activation of PLCγ1 and production of InsP_3_. InsP_3_ binds to and opens Ca^2+^ permeable InsP_3_ receptors (InsP_3_R) in the ER, resulting in the release of Ca^2+^ from ER stores (Lewis, 2001; Feske, 2007). Ca^2+^ release from the ER causes the activation of STIM 1 and 2, which oligomerize and translocate to ER-plasma membrane junctions. STIM1 and STIM2 bind to ORAI1, the pore-forming subunit of the CRAC channel, thereby mediating its opening and sustained Ca^2+^ influx. The subunit composition of the CRAC channel awaits further studies; both tetrameric and hexameric assemblies of ORAI1 subunits were proposed (Ji et al., 2008; Mignen et al., 2008; Penna et al., 2008; Maruyama et al., 2009; Hou et al., 2012). TRPM2 is a Ca^2+^ permeable cation channel that can be activated by cADPR and NAADP in human T cells (Beck et al., 2006). Increased cADPR levels after TCR stimulation (Guse et al., 1999) activate SOCE by releasing Ca^2+^ from the ER through RyR channels and potentially activate TRPM2 channels directly. TRPM7 is a non-selective cation channel implicated in Mg^2+^ homeostasis in T cells; whether its ability to conduct Ca^2+^ contributes to T cell function and how it is activated by TCR stimulation is not understood. The L-type Ca_v_ channels Ca_v_1.2, Ca_v_1.3, and Ca_v_1.4, which mediate depolarization-dependent Ca^2+^ influx in excitable cells including neurons may contribute to Ca^2+^ influx in T cells but their activation mechanism is unknown and their current properties are not well defined. P2X receptors are non-selective Ca^2+^ channels activated by extracellular ATP. Several homologs, P2X1, P2X4, and P2X7, were reported to mediate Ca^2+^ influx in T cells *in vitro*.

The essential role of CRAC channels for T cell function and adaptive immunity is best illustrated by patients with loss-of-function or null mutations in *ORAI1* or *STIM1* genes, whose T cells lack CRAC channel function and SOCE (Partiseti et al., 1994; Le Deist et al., 1995; Feske et al., 1996; McCarl et al., 2009; Picard et al., 2009; Feske, 2011; Fuchs et al., 2012). CRAC channel-deficient T cells proliferate poorly *in vitro* and have a profound defect in the production of cytokines such as IFNγ, TNFα, IL-2, and IL-17. Similar defects are found in CD4^+^ and CD8^+^ T cells from *Stim1*^−/−^, *Orai1*^−/−^, and *Orai1^R91W^* knock-in mice (Gwack et al., 2008; Beyersdorf et al., 2009; McCarl et al., 2010). SOCE-deficient T cells were found to be more resistant to apoptotic cell death and showed migration defects *in vitro* and *in vivo* (Ma et al., 2010; Kim et al., 2011; Greenberg et al., 2013) (and Stefan Feske unpublished data). Interestingly, SOCE is dispensable for the development and selection of conventional TCRαβ^+^ CD4^+^ and CD8^+^ T cells in SOCE-deficient patients and mice. However, their T cell function is severely compromised *in vivo*, apparent in absent delayed type hypersensitivity (DTH) responses to recall antigens in patients and mice (Le Deist et al., 1995; Feske et al., 1996; McCarl et al., 2010) and impaired skin allograft rejection in *Orai1^R93W^* knock-in mice (McCarl et al., 2010). Most importantly, impaired T cell function in ORAI1 and STIM1-deficient patients results in recurrent and chronic infections with a wide spectrum of viral, bacterial and fungal pathogens (Partiseti et al., 1994; Le Deist et al., 1995; Feske et al., 1996; McCarl et al., 2009; Picard et al., 2009; Byun et al., 2010; Feske, 2010; Fuchs et al., 2012).

Besides immunity to infection, CRAC channels in T cells regulate immunological tolerance and inflammation. CD4^+^ T cells from mice lacking ORAI1 or STIM1 function showed strongly impaired expression of proinflammatory cytokines such as IFN-γ and IL-17 (Ma et al., 2010; McCarl et al., 2010). Importantly, these mice were resistant to T cell-mediated intestinal and CNS inflammation in animal models of colitis and multiple sclerosis. Complete absence of CRAC channel function in mice with T cell-specific deletion of *Stim1* and *Stim2* genes, in addition, results in impaired development and function of Foxp3^+^ regulatory T (Treg) cells (Oh-Hora et al., 2008). As a result, STIM1/2-deficient mice over time develop severe myelo-lymphoproliferative disease with lymphadenopathy, splenomegaly, and pulmonary inflammation (Oh-Hora et al., 2008). Intriguingly, these mice show an exocrine gland autoimmune disease resembling Sjogren’s syndrome in humans (Cheng et al., 2012). Reduced numbers of Treg cells are also found in ORAI1*-* and STIM1-deficient patients (Picard et al., 2009) (and unpublished data), most of which suffer from autoimmune thrombocytopenia and hemolytic anemia due to autoantibodies against erythrocytes and platelets (Feske, 2011). The complete lack of SOCE in STIM1/2-deficient mice not only impaired the development of Treg cells but also that of natural killer T (NKT) cells and TCRαβ^+^ CD8αα^+^ intraepithelial lymphocytes (IEL) in the gut (Oh-Hora et al., 2013). These findings indicate that low to moderate SOCE is sufficient for the postselection maturation of agonist-selected T cells (Treg cells, NKT cells, IEL), whereas strong SOCE is required for the proinflammatory function of Th1 and Th17 cells.

Transient receptor potential channels belong to a large family of ion channels, which conduct monovalent and divalent cations including Ca^2+^ (Nilius and Owsianik, 2011). Before the discovery of ORAI1 as the CRAC channel, several TRPC channels were proposed to mediate Ca^2+^ influx in T cells. However, a significant role of TRPC channels in Ca^2+^ influx and T cell mediated immune function has not been established. By contrast, TRPM7 is essential for T cell development as mice with T cell-specific deletion of *Trpm7* had a severe block in T cell development at the CD4^−^CD8^−^ double negative stage (Jin et al., 2008). This is the most profound effect of any ion channel on lymphocyte development demonstrated so far. TRPM7 is Mg^2+^ permeable and widely considered to regulate cellular Mg^2+^ homeostasis. However, T cells from *Trpm7*^−*/*−^ mice had normal Mg^2+^ influx and total Mg^2+^ levels, raising the question whether impaired T cell development is caused by dysregulated Mg^2+^ homeostasis or rather by impaired influx of other cations including Ca^2+^ which TRPM7 is able to conduct as well. Another TRP channel, TRPM2 is a non-selective, Ca^2+^ permeable cation channel and in human T cells, TRPM2 can be activated by a variety of intracellular agonists including adenosine diphosphate ribose (ADPR), cyclic ADPR (cADPR), and Nicotinic acid adenine dinucleotide phosphate (NAADP) (Beck et al., 2006). TCR stimulation was reported to result in increased intracellular cADPR levels and Ca^2+^ release from the ER through ryanodine receptors (RyR) (Guse et al., 1999), thereby initiating SOCE; alternatively, elevated cADPR levels could directly activate TRPM2 channels. However, the physiological function of TRPM2 channels in T cells is unknown. It is intriguing to speculate that TRPM2 may be involved in inflammatory T cell responses similar to their role in CXCL2 chemokine expression and NADPH oxidase function in monocytes (Yamamoto et al., 2008) and phagocytes (Di et al., 2011).

Voltage-gated Ca^2+^ (Ca_v_) channels are highly Ca^2+^ selective channels that play an important role in Ca^2+^ influx and the function of electrically excitable cells such as neurons following cell depolarization (Tsien et al., 1987). In T cells, several members of the L-type family of Ca_V_ channels (Ca_V_1) were reported to be expressed but their contribution to Ca^2+^ influx has remained controversial (Hogan et al., 2010). Recent studies showed that genetic deletion of Ca_V_1.4 in mouse T cells and knockdown of Cav1.2 and Cav1.3 in human T cells attenuates TCR-induced Ca^2+^ influx (Cabral et al., 2010; Omilusik et al., 2011). Similarly, mutation of the regulatory β3 and β4 subunits of Ca_V_1 channels in mice results in reduced Ca^2+^ influx and impaired IL-4, IFNγ, and TNFα production in CD4^+^ and CD8^+^ T cells following TCR stimulation (Badou et al., 2006; Jha et al., 2009). CD8^+^ T cells lacking functional β3 regulatory subunits or Cav1.4 channels were more susceptible to apoptosis (Jha et al., 2009; Omilusik et al., 2011). Cav1.4-deficient mice also showed reduced cytotoxic function of CD8^+^ T cells *in vitro* and impaired CD8^+^ T cell responses to infection with *Listeria monocytogenes* *in vivo* (Omilusik et al., 2011). Despite these intriguing findings, the pathways by which TCR signaling activates Ca_V_1 channels are unknown. In contrast to excitable cells, depolarization of T cells fails to open Ca_V_1 channels and mediate Ca^2+^ influx. It has been speculated that Ca_V_1 channels in T cells are activated by an alternative, voltage-independent mechanism, but the nature of this mechanism remains to be elucidated. In addition, native Ca_V_1 channel-like Ca^2+^ currents have so far been reported only once despite efforts by numerous labs and appear to be restricted to naïve CD4^+^ and CD8^+^ T cells (Omilusik et al., 2011). Intriguingly, these currents were abolished in T cells from *Cav1.4*^−/−^ mice. Future studies will need to investigate the electrophysiological properties of Ca_V_1 channels in T cells, clarify the molecular mechanisms that regulate their activation, and investigate the role of Ca_V_1 channels in adaptive immunity.

P2X receptors are not, unlike the Ca^2+^ channels discussed above, activated by TCR stimulation but by extracellular ATP. Three P2X receptors, P2X1, P2X4 and P2X7 (Yip et al., 2009; Woehrle et al., 2010), were reported to mediate Ca^2+^ influx in T cells. The opening of P2X7 causes Ca^2+^ influx and activation of enzymes such as calcineurin, resulting in T cell proliferation (Baricordi et al., 1996) and IL-2 production (Adinolfi et al., 2005; Woehrle et al., 2010). Inhibition of P2X1, P2X4, and P2X7 function by RNAi or chemical antagonists attenuated Ca^2+^ influx and IL-2 production in Jurkat cells and human CD4^+^ T cells *in vitro* (Yip et al., 2009; Woehrle et al., 2010). A Ca^2+^ dependent role for individual P2X receptors in T cell immunity *in vivo*, however, is not well established. Using *P2*X*7*^−/−^ mice, one study found that P2X7 is proinflammatory in T cells by promoting the differentiation and function of Th17 cells and inhibiting the stability of Treg cells (Schenk et al., 2011). The role of P2X7 in T cell-mediated autoimmunity, however, is ambiguous as both increased (Chen and Brosnan, 2006) and decreased (Sharp et al., 2008) CNS inflammation were observed in *P2*X*7*^−/−^ mice when analyzed in animal models of multiple sclerosis. In addition, it is unclear whether the main function of P2X7 receptors in T cells is to mediate Ca^2+^ influx. At the high ATP concentrations (∼1 mM) used to measure Ca^2+^ influx in most studies, P2X7 channels form a large pore (Junger, 2011) that is permeable to a variety of anorganic and organic cations (Chused et al., 1996; Adriouch et al., 2002). Similarly high ATP concentrations are used to activate inflammasomes in innate immune cells, in which P2X7 channels are thought to mediate K^+^ efflux and thereby production of IL-1β (Ferrari et al., 2006; Tschopp and Schroder, 2010). Future studies need to evaluate if P2X7 receptors modulate T cell function through Ca^2+^ influx or other mechanisms. P2X1 and P2X4 conduct Ca^2+^ more selectively and open at lower (micromolar) ATP concentrations (Junger, 2011). However, P2X1 and P2X4-deficient mice have no reported immunological phenotype (Mulryan et al., 2000; Yamamoto et al., 2006) and their role in T cell immunity *in vivo* remains poorly understood.

Ca^2+^ signals have long been recognized as essential for T cell function and several channels may contribute to Ca^2+^ influx in T cells. Whereas the role of CRAC channels to T cell function and adaptive immunity is well documented by findings in ORAI1 and STIM1-deficient patients and mice, the contributions of TRP, Ca_V_1, and P2X receptor channels remain to be more clearly defined. These channels could contribute to Ca^2+^ influx in specific T cell subsets, at distinct stages of T cell development or following stimuli other than TCR engagement. A better understanding of the contributions of different Ca^2+^ influx pathways in T cells will be essential to define potential drug targets for the modulation of T cell function in a variety of diseases caused by aberrant T cell function.

## References

[B1] Adinolfi E. Callegari M. G. Ferrari D. Bolognesi C. Minelli M. Wieckowski M. R. (2005). Basal activation of the P2×7 ATP receptor elevates mitochondrial calcium and potential, increases cellular ATP levels, and promotes serum-independent growth. Mol. Biol. Cell 16, 3260–3272 10.1091/mbc.E04-11-1025 15901833 PMC1165409

[B2] Adriouch S. Dox C. Welge V. Seman M. Koch-Nolte F. Haag F. (2002). Cutting edge: a natural P451L mutation in the cytoplasmic domain impairs the function of the mouse P2×7 receptor. J. Immunol. 169, 4108–4112 12370338 10.4049/jimmunol.169.8.4108

[B3] Badou A. Jha M. K. Matza D. Mehal W. Z. Freichel M. Flockerzi V. (2006). Critical role for the beta regulatory subunits of Cav channels in T lymphocyte function. Proc. Natl. Acad. Sci. U.S.A. 103, 15529–15534 10.1073/pnas.0607262103 17028169 PMC1622857

[B4] Baricordi O. R. Ferrari D. Melchiorri L. Chiozzi P. Hanau S. Chiari E. (1996). An ATP-activated channel is involved in mitogenic stimulation of human T lymphocytes. Blood 87, 682–690 8555491

[B5] Beck A. Kolisek M. Bagley L. A. Fleig A. Penner R. (2006). Nicotinic acid adenine dinucleotide phosphate and cyclic ADP-ribose regulate TRPM2 channels in T lymphocytes. FASEB J. 20, 962–964 10.1096/fj.05-5538fje 16585058

[B6] Beyersdorf N. Braun A. Vogtle T. Varga-Szabo D. Galdos R. R. Kissler S. (2009). STIM1-independent T cell development and effector function in vivo. J. Immunol. 182, 3390–3397 10.4049/jimmunol.0802888 19265116

[B7] Byun M. Abhyankar A. Lelarge V. Plancoulaine S. Palanduz A. Telhan L. (2010). Whole-exome sequencing-based discovery of STIM1 deficiency in a child with fatal classic Kaposi sarcoma. J. Exp. Med. 207, 2307–2312 10.1084/jem.20101597 20876309 PMC2964585

[B8] Cabral M. D. Paulet P. E. Robert V. Gomes B. Renoud M. L. Savignac M. (2010). Knocking down Cav1 calcium channels implicated in Th2 cell activation prevents experimental asthma. Am. J. Respir. Crit. Care Med. 181, 1310–1317 10.1164/rccm.200907-1166OC 20167851

[B9] Cahalan M. D. Chandy K. G. (2009). The functional network of ion channels in T lymphocytes. Immunol. Rev. 231, 59–87 10.1111/j.1600-065X.2009.00816.x 19754890 PMC3133616

[B10] Chen L. Brosnan C. F. (2006). Exacerbation of experimental autoimmune encephalomyelitis in P2×7R-/- mice: evidence for loss of apoptotic activity in lymphocytes. J. Immunol. 176, 3115–3126 16493071 10.4049/jimmunol.176.5.3115

[B11] Cheng K. T. Alevizos I. Liu X. Swaim W. D. Yin H. Feske S. (2012). STIM1 and STIM2 protein deficiency in T lymphocytes underlies development of the exocrine gland autoimmune disease, Sjogren’s syndrome. Proc. Natl. Acad. Sci. U.S.A. 109, 14544–14549 10.1073/pnas.1113363109 22904194 PMC3437853

[B12] Chused T. M. Apasov S. Sitkovsky M. (1996). Murine T lymphocytes modulate activity of an ATP-activated P2Z-type purinoceptor during differentiation. J. Immunol. 157, 1371–1380 8759716

[B13] Di A. Gao X. P. Qian F. Kawamura T. Han J. Hecquet C. (2011). The redox-sensitive cation channel TRPM2 modulates phagocyte ROS production and inflammation. Nat. Immunol. 13, 29–34 10.1038/ni.2171 22101731 PMC3242890

[B14] Ferrari D. Pizzirani C. Adinolfi E. Lemoli R. M. Curti A. Idzko M. (2006). The P2×7 receptor: a key player in IL-1 processing and release. J. Immunol. 176, 3877–3883 16547218 10.4049/jimmunol.176.7.3877

[B15] Feske S. (2007). Calcium signalling in lymphocyte activation and disease. Nat. Rev. Immunol. 7, 690–702 10.1038/nri2152 17703229

[B16] Feske S. (2010). CRAC channelopathies. Pflugers Arch. 460, 417–435 10.1007/s00424-009-0777-5 20111871 PMC2885504

[B17] Feske S. (2011). Immunodeficiency due to defects in store-operated calcium entry. Ann. N. Y. Acad. Sci. 1238, 74–90 10.1111/j.1749-6632.2011.06240.x 22129055 PMC3774594

[B18] Feske S. Gwack Y. Prakriya M. Srikanth S. Puppel S. H. Tanasa B. (2006). A mutation in Orai1 causes immune deficiency by abrogating CRAC channel function. Nature 441, 179–185 10.1038/nature04702 16582901

[B19] Feske S. Muller J. M. Graf D. Kroczek R. A. Drager R. Niemeyer C. (1996). Severe combined immunodeficiency due to defective binding of the nuclear factor of activated T cells in T lymphocytes of two male siblings. Eur. J. Immunol. 26, 2119–2126 10.1002/eji.1830260924 8814256

[B20] Feske S. Skolnik E. Y. Prakriya M. (2012). Ion channels and transporters in lymphocyte function and immunity. Nat. Rev. Immunol. 12, 532–547 10.1038/nri3233 22699833 PMC3670817

[B21] Fuchs S. Rensing-Ehl A. Speckmann C. Bengsch B. Schmitt-Graeff A. Bondzio I. (2012). Antiviral and regulatory T cell immunity in a patient with stromal interaction molecule 1 deficiency. J. Immunol. 188, 1523–1533 10.4049/jimmunol.1102507 22190180 PMC3262903

[B22] Greenberg M. L. Yu Y. Leverrier S. Zhang S. L. Parker I. Cahalan M. D. (2013). Orai1 function is essential for T cell homing to lymph nodes. J. Immunol. 190, 3197–3206 10.4049/jimmunol.1202212 23455504 PMC3608704

[B23] Guse A. H. Da Silva C. P. Berg I. Skapenko A. L. Weber K. Heyer P. (1999). Regulation of calcium signalling in T lymphocytes by the second messenger cyclic ADP-ribose. Nature 398, 70–73 10.1038/18024 10078531

[B24] Gwack Y. Srikanth S. Oh-Hora M. Hogan P. G. Lamperti E. D. Yamashita M. (2008). Hair loss and defective T- and B-cell function in mice lacking ORAI1. Mol. Cell. Biol. 28, 5209–5222 10.1128/MCB.00360-08 18591248 PMC2519726

[B25] Hogan P. G. Lewis R. S. Rao A. (2010). Molecular basis of calcium signaling in lymphocytes: STIM and ORAI. Annu. Rev. Immunol. 28, 491–533 10.1146/annurev.immunol.021908.132550 20307213 PMC2861828

[B26] Hoth M. Penner R. (1992). Depletion of intracellular calcium stores activates a calcium current in mast cells. Nature 355, 353–356 10.1038/355353a0 1309940

[B27] Hou X. Pedi L. Diver M. M. Long S. B. (2012). Crystal structure of the calcium release-activated calcium channel Orai. Science 338, 1308–1313 10.1126/science.1228757 23180775 PMC3695727

[B28] Jha M. K. Badou A. Meissner M. McRory J. E. Freichel M. Flockerzi V. (2009). Defective survival of naive CD8+ T lymphocytes in the absence of the beta3 regulatory subunit of voltage-gated calcium channels. Nat. Immunol. 10, 1275–1282 10.1038/ni.1793 19838200 PMC2785134

[B29] Ji W. Xu P. Li Z. Lu J. Liu L. Zhan Y. (2008). Functional stoichiometry of the unitary calcium-release-activated calcium channel. Proc. Natl. Acad. Sci. U.S.A. 105, 13668–13673 10.1073/pnas.0806499105 18757751 PMC2533247

[B30] Jin J. Desai B. N. Navarro B. Donovan A. Andrews N. C. Clapham D. E. (2008). Deletion of Trpm7 disrupts embryonic development and thymopoiesis without altering Mg2+ homeostasis. Science 322, 756–760 10.1126/science.1163493 18974357 PMC2605283

[B31] Junger W. G. (2011). Immune cell regulation by autocrine purinergic signalling. Nat. Rev. Immunol. 11, 201–212 10.1038/nri2938 21331080 PMC4209705

[B32] Kim K. D. Srikanth S. Yee M. K. Mock D. C. Lawson G. W. Gwack Y. (2011). ORAI1 deficiency impairs activated T cell death and enhances T cell survival. J. Immunol. 187, 3620–3630 10.4049/jimmunol.1101654 21873530 PMC3178683

[B33] Le Deist F. Hivroz C. Partiseti M. Thomas C. Buc H. A. Oleastro M. (1995). A primary T-cell immunodeficiency associated with defective transmembrane calcium influx. Blood 85, 1053–1062 7531512

[B34] Lewis R. S. (2001). Calcium signaling mechanisms in T lymphocytes. Annu. Rev. Immunol. 19, 497–521 10.1146/annurev.immunol.19.1.497 11244045

[B35] Lewis R. S. Cahalan M. D. (1989). Mitogen-induced oscillations of cytosolic Ca2+ and transmembrane Ca2+ current in human leukemic T cells. Cell Regul. 1, 99–112 2519622 10.1091/mbc.1.1.99 PMC361429

[B36] Ma J. McCarl C. A. Khalil S. Luthy K. Feske S. (2010). T-cell-specific deletion of STIM1 and STIM2 protects mice from EAE by impairing the effector functions of Th1 and Th17 cells. Eur. J. Immunol. 2010, 9 10.1002/eji.201040614 PMC3517124 21061435

[B37] Maruyama Y. Ogura T. Mio K. Kato K. Kaneko T. Kiyonaka S. (2009). Tetrameric Orai1 is a teardrop-shaped molecule with a long, tapered cytoplasmic domain. J. Biol. Chem. 284, 13676–13685 10.1074/jbc.M109.066712 19289460 PMC2679469

[B38] McCarl C. A. Khalil S. Ma J. Oh-Hora M. Yamashita M. Roether J. (2010). Store-operated Ca2+ entry through ORAI1 is critical for T cell-mediated autoimmunity and allograft rejection. J. Immunol. 185, 5845–5858 10.4049/jimmunol.1001796 20956344 PMC2974040

[B39] McCarl C. A. Picard C. Khalil S. Kawasaki T. Rother J. Papolos A. (2009). ORAI1 deficiency and lack of store-operated Ca2+ entry cause immunodeficiency, myopathy, and ectodermal dysplasia. J. Allergy Clin. Immunol. 124, e1317 10.1016/j.jaci.2009.10.007 PMC2829767 20004786

[B40] McNally B. A. Somasundaram A. Yamashita M. Prakriya M. (2012). Gated regulation of CRAC channel ion selectivity by STIM1. Nature 482, 241–245 22278058 10.1038/nature10752 PMC3276717

[B41] Mignen O. Thompson J. L. Shuttleworth T. J. (2008). Orai1 subunit stoichiometry of the mammalian CRAC channel pore. J. Physiol. (Lond.) 586, 419–425 10.1113/jphysiol.2007.147249 18006576 PMC2375595

[B42] Mulryan K. Gitterman D. P. Lewis C. J. Vial C. Leckie B. J. Cobb A. L. (2000). Reduced vas deferens contraction and male infertility in mice lacking P2×1 receptors. Nature 403, 86–89 10.1038/47495 10638758

[B43] Nilius B. Owsianik G. (2011). The transient receptor potential family of ion channels. Genome Biol. 12, 218 10.1186/gb-2011-12-3-218 21401968 PMC3129667

[B44] Oh-Hora M. Komatsu N. Pishyareh M. Feske S. Hori S. Taniguchi M. (2013). Agonist-selected T cell development requires strong T cell receptor signaling and store-operated calcium entry. Immunity 38, 1–15 10.1016/j.immuni.2013.01.005 23499491 PMC3669219

[B45] Oh-Hora M. Yamashita M. Hogan P. G. Sharma S. Lamperti E. Chung W. (2008). Dual functions for the endoplasmic reticulum calcium sensors STIM1 and STIM2 in T cell activation and tolerance. Nat. Immunol. 9, 432–443 10.1038/ni1574 18327260 PMC2737533

[B46] Omilusik K. Priatel J. J. Chen X. Wang Y. T. Xu H. Choi K. B. (2011). The Ca(v)1.4 calcium channel is a critical regulator of T cell receptor signaling and naive T cell homeostasis. Immunity 35, 349–360 10.1016/j.immuni.2011.07.011 21835646

[B47] Parekh A. B. Penner R. (1997). Store depletion and calcium influx. Physiol. Rev. 77, 901–930 9354808 10.1152/physrev.1997.77.4.901

[B48] Partiseti M. Le Deist F. Hivroz C. Fischer A. Korn H. Choquet D. (1994). The calcium current activated by T cell receptor and store depletion in human lymphocytes is absent in a primary immunodeficiency. J. Biol. Chem. 269, 32327–32335 7798233

[B49] Penna A. Demuro A. Yeromin A. V. Zhang S. L. Safrina O. Parker I. (2008). The CRAC channel consists of a tetramer formed by Stim-induced dimerization of Orai dimers. Nature 456, 116–120 10.1038/nature07338 18820677 PMC2597643

[B50] Picard C. McCarl C. A. Papolos A. Khalil S. Luthy K. Hivroz C. (2009). STIM1 mutation associated with a syndrome of immunodeficiency and autoimmunity. N. Engl. J. Med. 360, 1971–1980 10.1056/NEJMoa0900082 19420366 PMC2851618

[B51] Prakriya M. Feske S. Gwack Y. Srikanth S. Rao A. Hogan P. G. (2006). Orai1 is an essential pore subunit of the CRAC channel. Nature 443, 230–233 10.1038/nature05122 16921383

[B52] Prakriya M. Lewis R. S. (2003). CRAC channels: activation, permeation, and the search for a molecular identity. Cell Calcium 33, 311–321 10.1016/S0143-4160(03)00045-9 12765678

[B53] Schenk U. Frascoli M. Proietti M. Geffers R. Traggiai E. Buer J. (2011). ATP inhibits the generation and function of regulatory T cells through the activation of purinergic P2X receptors. Sci. Signal. 4, ra12 10.1126/scisignal.2001270 21364186

[B54] Sharp A. J. Polak P. E. Simonini V. Lin S. X. Richardson J. C. Bongarzone E. R. (2008). P2×7 deficiency suppresses development of experimental autoimmune encephalomyelitis. J. Neuroinflammation 5, 33 10.1186/1742-2094-5-33 18691411 PMC2518548

[B55] Shaw P. J. Qu B. Hoth M. Feske S. (2012). Molecular regulation of CRAC channels and their role in lymphocyte function. Cell. Mol. Life Sci. [Epub ahead of print]. 10.1007/s00018-012-1175-2 23052215 PMC3553310

[B56] Tschopp J. Schroder K. (2010). NLRP3 inflammasome activation: the convergence of multiple signalling pathways on ROS production? Nat. Rev. Immunol. 10, 210–215 10.1038/nri2725 20168318

[B57] Tsien R. W. Hess P. McCleskey E. W. Rosenberg R. L. (1987). Calcium channels: mechanisms of selectivity, permeation, and block. Annu. Rev. Biophys. Biophys. Chem. 16, 265–290 10.1146/annurev.bb.16.060187.001405 2439098

[B58] Vig M. Beck A. Billingsley J. M. Lis A. Parvez S. Peinelt C. (2006a). CRACM1 multimers form the ion-selective pore of the CRAC channel. Curr. Biol. 16, 2073–2079 10.1016/j.cub.2006.08.085 16978865 PMC5685803

[B59] Vig M. Peinelt C. Beck A. Koomoa D. L. Rabah D. Koblan-Huberson M. (2006b). CRACM1 is a plasma membrane protein essential for store-operated Ca2+ entry. Science 312, 1220–1223 10.1126/science.1127883 16645049 PMC5685805

[B60] Woehrle T. Yip L. Elkhal A. Sumi Y. Chen Y. Yao Y. (2010). Pannexin-1 hemichannel-mediated ATP release together with P2×1 and P2×4 receptors regulate T-cell activation at the immune synapse. Blood 116, 3475–3484 10.1182/blood-2010-04-277707 20660288 PMC2981474

[B61] Yamamoto K. Sokabe T. Matsumoto T. Yoshimura K. Shibata M. Ohura N. (2006). Impaired flow-dependent control of vascular tone and remodeling in P2×4-deficient mice. Nat. Med. 12, 133–137 10.1038/nm1338 16327800

[B62] Yamamoto S. Shimizu S. Kiyonaka S. Takahashi N. Wajima T. Hara Y. (2008). TRPM2-mediated Ca2+ influx induces chemokine production in monocytes that aggravates inflammatory neutrophil infiltration. Nat. Med. 14, 738–747 10.1038/nm1758 18542050 PMC2789807

[B63] Yeromin A. V. Zhang S. L. Jiang W. Yu Y. Safrina O. Cahalan M. D. (2006). Molecular identification of the CRAC channel by altered ion selectivity in a mutant of Orai. Nature 443, 226–229 10.1038/nature05108 16921385 PMC2756048

[B64] Yip L. Woehrle T. Corriden R. Hirsh M. Chen Y. Inoue Y. (2009). Autocrine regulation of T-cell activation by ATP release and P2×7 receptors. FASEB J. 23, 1685–1693 10.1096/fj.08-126458 19211924 PMC2718802

[B65] Zhang S. L. Yeromin A. V. Zhang X. H. Yu Y. Safrina O. Penna A. (2006). Genome-wide RNAi screen of Ca(2+) influx identifies genes that regulate Ca(2+) release-activated Ca(2+) channel activity. Proc. Natl. Acad. Sci. U.S.A. 103, 9357–9362 10.1073/pnas.0608556103 16751269 PMC1482614

[B66] Zweifach A. Lewis R. S. (1993). Mitogen-regulated Ca2+ current of T lymphocytes is activated by depletion of intracellular Ca2+ stores. Proc. Natl. Acad. Sci. U.S.A. 90, 6295–6299 10.1073/pnas.90.13.6295 8392195 PMC46915

